# Supplementary material for: Young Adults with Higher Salt Intake Have Inferior Hydration Status: A Cross-Sectional Study
Source: Nutrients. 2022 Jan 11;14(2):287. doi: 10.3390/nu14020287 (PMC8778661; doi:10.3390/nu14020287)
Supplement: Supplementary file 1 [file nutrients-14-00287-s001.zip › nutrients-1471719-Supplementary materials.pdf]

Supplementary material 1 The temperature and humidity of study days

	Indoors		Outdoors	
	Temperature (℃)	Humidity (%)	Temperature (℃)	Humidity (%)
Sunday	19.9	43	18.1	37
Monday	23.0	48	22.4	41
Tuesday	23.3	31	24.0	29
Wednesday	21.5	48	17.9	42
Thursday	21.5	40	21.0	36
Friday	22.2	35	19.2	35
Saturday	21.2	34	22.6	31
